# Autoantibodies Affect Brain Density Reduction in Nonneuropsychiatric Systemic Lupus Erythematosus Patients

**DOI:** 10.1155/2015/920718

**Published:** 2015-05-18

**Authors:** Jian Xu, Yuqi Cheng, Aiyun Lai, Zhaoping Lv, Robert A. A. Campbell, Hongjun Yu, Chunrong Luo, Baoci Shan, Lin Xu, Xiufeng Xu

**Affiliations:** ^1^Department of Rheumatology and Immunology, First Affiliated Hospital of Kunming Medical University, Kunming 650032, China; ^2^Department of Psychiatry, First Affiliated Hospital of Kunming Medical University, Kunming 650032, China; ^3^Department of Neuroscience, Cold Spring Harbor Laboratory, New York, NY 11724, USA; ^4^Magnetic Resonance Imaging Center, First Hospital of Kunming City, Kunming 650011, China; ^5^Key Laboratory of Nuclear Analysis, Institute of High Energy Physics, Chinese Academy of Sciences, Beijing 100049, China; ^6^Key Laboratory of Animal Models and Human Disease Mechanisms, Chinese Academy of Sciences, Kunming 650223, China

## Abstract

This study explores the relationship between autoantibodies and brain density reduction in SLE patients without major neuropsychiatric manifestation (NPSLE). Ninety-five NPSLE patients without obvious cerebral deficits, as determined by conventional MRI, as well as 89 control subjects, underwent high-resolution structural MRI. Whole-brain density of grey matter (GMD) and white matter (WMD) were calculated for each individual, and correlations between the brain density, symptom severity, immunosuppressive agent (ISA), and autoantibody levels were assessed. The GMD and WMD of the SLE group decreased compared to controls. GMD was negatively associated with SLE activity. The WMD of patients who received ISA treatment were higher than that in the patients who did not. The WMD of patients with anticardiolipin (ACL) or anti-SSB/La antibodies was lower than in patients without these antibodies, while the GMD was lower in patients with anti-SM or anti-U1RNP antibodies. Thus, obvious brain atrophy can occur very early even before the development of significant symptoms and specific autoantibodies might contribute to the reduction of GMD or WMD in NPSLE patients. However, ISAs showed protective effects in minimizing GMD and WMD reduction. The presence of these specific autoantibodies might help identify early brain damage in NPSLE patients.

## 1. Introduction

Systemic lupus erythematosus (SLE) is an autoimmune disease involving almost all of the organ systems. Central nervous system (CNS) involvement is typical during the course of SLE [1, 2]. Brain atrophy has long been reported in SLE using neuroimaging techniques [3] and often correlates with clinical manifestations, even in patients without clear CNS signs and symptoms [4]. Magnetic resonance imaging (MRI) is widely used to detect brain anatomy abnormalities, including cerebral atrophy [3, 5, 6]. Although MRIs are widely used to evaluate CNS involvement in SLE, conventional or anatomical MRI findings are sometimes nonspecific or negative [7] in patients with and without neuropsychiatric SLE (NPSLE). Many patients with mood or cognitive disorders have been identified as normal according to conventional MRI. There is evidence that abnormal WM microstructures may be found in non-NPSLE patients or in patients with an apparently normal brain structure [8], suggesting that there may be microstructural abnormalities before obvious CNS manifestations appear. Although important for clinical evaluation, the discrimination of mild structural abnormalities in these patients is difficult. If a subclinical involvement of the brain microstructure could be identified before the emergence of clear neuropsychiatric symptoms, earlier intervention could be initiated, potentially preventing progressive brain injury.

The pathogenesis of CNS involvement in SLE patients remains unclear. Various autoantibodies have been implicated in the pathogenesis of NPSLE, including anticardiolipin antibodies (ACL) [9]. Because they are prothrombotic, ACL antibodies may cause cerebral infarctions and correlate with focal neurological syndromes [10]. Associations between ACL antibodies and nonfocal neuropsychiatric manifestations have also been reported [11]. Antiribosomal P-protein (P0) antibodies recognize specific proteins on ribosomes. P0 antibodies detected in blood have been associated with psychosis in some studies [12]. Although these autoantibodies are considered to play important roles in the etiology of SLE, few studies have focused on the relationship between the autoantibodies and structural brain damage. Only a VBM study reported that the presence of antiphospholipid antibodies was associated with white and gray matter loss [13]. However, the role of antibodies in the pathogenesis of neuropsychiatric symptoms in patients without conventional MRI abnormalities remains unclear.

Here, we evaluate whether there is microstructural brain atrophy in a relatively large sample of SLE patients without NPSLE who were diagnosed as normal by conventional MRI. Another objective of this study was to explore the potential association between these brain abnormalities and the presence of specific autoantibodies.

## 2. Material and Methods 

### 2.1. Subjects

SLE patients treated in the in-patient or out-patient facilities of the Rheumatology and Immunology Department of the First Affiliated Hospital of Kunming Medical University (from September 2009 to November 2011) and from the Chinese SLE Treatment and Research Group (CSTAR) member units were recruited in this study. All of the participants were studied via a standardized protocol and were followed by the same investigator throughout the course of the study. Prior to entry into the study, each participant provided written informed consent after receiving a complete description of the study. This research was approved by the Institutional Review Board of Kunming Medical University, Yunnan Province, China (ClinicalTrials.gov: NCT00703742).

The following were the inclusion criteria: (1) patients diagnosed as having SLE by four or more criteria from the 1997 revised American College of Rheumatology (ACR) criteria for the classification of SLE [14]; (2) subjects between the ages of 16 and 50; and (3) subjects willing to attend this study and who gave written consent.

The exclusion criteria included the following: (1) patients fulfilling the ACR criteria for rheumatoid arthritis, systemic sclerosis, Sjögren syndrome (primary or secondary) or other connective tissue diseases, or drug-induced SLE; (2) patients with organic brain or neurological disorders that would disturb the structure or diffusion imaging of the brain (i.e., history of head trauma, Parkinson's disease, or seizures); (3) patients with major active CNS manifestations, such as an obviously disorganized behavior, psychiatric disorders, conscious disturbances, and neurological symptoms; (4) patients with a history of substance abuse; (5) patients who are pregnant or have any physical illness assessed by personal history; (6) patients unable to undergo MRI or with claustrophobia or a pacemaker; (7) patients with serious clinical conditions that could influence cerebral atrophy, such as a history of arterial hypertension, diabetes mellitus, stroke, or renal insufficiency; and (8) structural abnormities of the brain identified by a conventional T1 and T2 weighted MRI.

One-hundred three diagnosed SLE patients were interviewed. All 103 patients were tested using conventional and additional laboratory tests (thyroid function tests and renal function tests, etc.), disease activity scales, questionnaires, and MRI scans. Ninety-eight healthy controls (CTLs) were also recruited. Complete general physical and neurological examinations were given to all of the CTLs by an experienced rheumatologist and neurologist, respectively, to exclude major disorders or, especially, neurological problems. Psychiatric symptoms were screened by an experienced psychiatrist using the Structured Clinical Interview for DSM-IV, Nonpatient Version (SCID-NP). All of the participants were Han Chinese people and were right-handed.

### 2.2. Scales and Clinical Features of SLE Patients

Data on sex, age at disease onset, and disease duration were collected for each patient. Disease duration was defined as the period from the initial manifestation that was clearly attributable to SLE until the day of the MRI acquisition. All of the clinical manifestations and laboratory test findings were recorded according to the ACR criteria [14]. Disease activity was measured by the systemic lupus erythematosus disease activity index (SLEDAI), and cumulative SLE-related damage was determined by the Systemic Lupus International Collaborating Clinics/American College of Rheumatology Damage Index for Systemic Lupus Erythematosus (SLICC/ACRDI) [15] in all of the SLE patients at the time of the MRI. Active disease was defined as a SLEDAI score higher than 8 [16].

Data on the total dose of corticosteroids and immunosuppressive agents from the time of drug initiation until the study date (including previous and current treatment) were collected by a careful interview. The cumulative dose of the immunosuppressive agents was calculated by summing the daily dosages and multiplying by the number of treatment days. The total doses of oral and intravenous corticosteroids were calculated by conversion to equivalent doses of prednisone.

A complete neurological examination was given to all of the patients to exclude major neurological problems, such as stroke and seizure. Obviously disorganized behavior and psychiatric symptoms, such as illusion and delusion, might imply possible serious involvement of the brain. Therefore, the patients with these symptoms were also excluded. All of the participants were right-handed, as assessed by the Edinburgh Handed Inventory [17]. All of the clinical data were collected on MRI examination days by an experienced psychiatrist.

### 2.3. Autoantibody Detect

Ten autoantibodies from all of the patients were tested, including the SLE-characteristic antibodies antinuclear antibodies (ANA), anti-dsDNA, anti-SSA/Ro52 kD, anti-SSA/Ro60 kD, anti-SSB/La, anti-SM, and previously reported antibodies related to CNS damage, such as ACL, antihistone, anti-P0, antinucleosome, and anti-U1RNP antibodies. The ANA tests were assessed by indirect immunofluorescence using Hep-2 cells as the substrate (SCIMEDX Corporation, New Jersey, USA); the anti-double-stranded DNA antibodies were determined using* Crithidia luciliae* as the substrate (H&J Novomed Ltd., Beijing, China); the ACL tests were assessed by conventional ELISA (Aesku Diagnostics, Wendelsheim, Germany). IMTEC-ANA-LIA (IMTEC, Wiesbaden, Germany) is a line immunoassay (LIA) that detects antinuclear antibodies (dsDNA, antinucleosome, SmD1, P0, antihistones, U1-RNP, SSA/Ro60 kD, SSA/Ro52 kD, and SSB/La). The assay was performed according to the manufacturer's instructions. All of the autoantibody samples were collected on the MRI examination day.

### 2.4. Image-Acquisition

Image-acquisition was performed by an experienced neuroradiologist. MRI sequences were performed on all of the subjects with a 1.5-T clinical GE MRI scanner (Twinspeed, Milwaukee, WI, USA) equipped with a birdcage head coil. Supportive foam pads were used to minimize head motion. A rapid sagittal localizer scan was used to confirm alignment. Normal T1 and T2 MRI scans were taken to exclude obvious structural abnormalities. Of all of the 103 SLE patients who received MRI scans, eight patients were excluded due to structural abnormities of the brain that were identified by common T1 and T2 weighted MRIs (three for local infarction, three for ischemia, and two for a WM hyperintense signal near the caudate nucleus). The data from the remaining 95 patients were included in this study. Nine subjects from the CTL group were also excluded due to local ischemia. A set of three-dimensional volumetric structural MRI scans were performed on each subject using a fast spoiled gradient echo sequence (FSPGR) with the following parameters: TR/TE = 10.5/2 ms, matrix size = 256 × 256, thickness = 1.8 mm with no interslice gap, field of view = 240 mm, and flip angle = 90°. Whole-brain data were acquired in axial planes parallel to the anterior commissure-posterior commissure line, yielding 172 continuous slices, with individual thicknesses of 0.9 mm.

### 2.5. Data Preprocessing and VBM Statistical Analysis

The DICOM image data were processed via MRIcro software (version 1.40; http://www.mricro.com/). All of the data were analyzed via statistical parametric mapping (SPM5, Wellcome Department of Cognitive Neurology, London, UK; http://www.fil.ion.ucl.ac.uk/) and VBM5 (http://dbm.neuro.uni-jena.de/vbm/vbm5-for-spm5/) software based on Matlab 7.1 (The MathWorks, Inc., Natick, MA, USA). Each individual image was normalized and transformed into the standardized Montreal Neurological Institute (MNI) template and then resampled in the 2 × 2 × 2 mm dimensional scale. The normalized images were then segmented into grey matter (GM), white matter (WM), and cerebrospinal fluid. The unmodulated GM and WM images were separately smoothed to remove noise using a filter with a half-width half-maximum of 8 mm.

### 2.6. Analysis of the Mean Whole Brain Grey Matter Density (GMD) and Mean Whole Brain White Matter Density (WMD)

Initially, we used the standard GM and WM templates in SPM5 as the whole-brain GM and WM masks. Then, using the smoothed GM and WM images from each participant, the GMD and WMD were retrieved. Two-sample *t*-tests were performed to analyze the differences in GMD and WMD between the two groups, using SPSS version 17.0 (SPSS, Inc.). Correlation and partial correlation methods were used to analyze the correlations between the disease characteristics and the WM volume of clusters. Covariance analysis was performed to detect the effect of different therapies on the GMD and WMD. Finally, we used two-sample *t*-tests to determine whether there were any differences in the GMD and WMD between the patients with different autoantibodies.

## 3. Results

### 3.1. Demographic Data on SLE Patients and HCs

In total, 95 SLE and 89 CTL subjects were analyzed in this study. The mean age was 28.65 years [standard deviation (SD) = 7.51, range 16–48] for the SLE patients and 30.70 years (SD = 7.93, range 17–50) for HCs. There were no significant differences in age or sex between these two groups (Table 1).

### 3.2. Clinical, Laboratory, and Treatment Features

The disease duration in SLE patients ranged from 0.5 to 204 months (mean = 18.99 months, SD = 27.55). Forty-seven patients were newly diagnosed with SLE, and 56 patients had disease durations that were less than 12 months. The other 39 patients had disease durations 13–204 months. At the time of the MRI scanning, the mean SLEDAI score was 10.01 (SD = 6.45; range 0–30). Of the 95 SLE patients, 33 patients were positive for APL, 51 for antihistone antibody, 46 for anti-P0, 51 for anti-SSA/Ro52 kD, 61 for anti-SSA/Ro60 kD, 34 for anti-SSB/La, 38 for antinucleosome, 30 for anti-U1RNP, 45 for anti-SM, and 63 for anti-dsDNA. According to the SLICC, fifteen patients had a score of 1 (ten cases had proteinuria >3.5 gm/24 hours and five cases had cutaneous small vessel vasculitis in a terminal finger or minor tissue loss). The remaining 80 patients were without serious organic impairment; their SLICC score was 0. The mean SLICC score for all of the patients was 0.158 (SD = 0.367; range 0-1). Of the 95 patients, 55 were treated with immunosuppressive agents (ISA) [17 with cyclophosphamide (CTX), 32 with hydroxychloroquine (HCQ), and 6 with both]. The other 41 patients were never treated with immunosuppressive agents (Table 1).

### 3.3. GMD/WMD Differences between the SLE and CTL Groups

**Figure 1:**
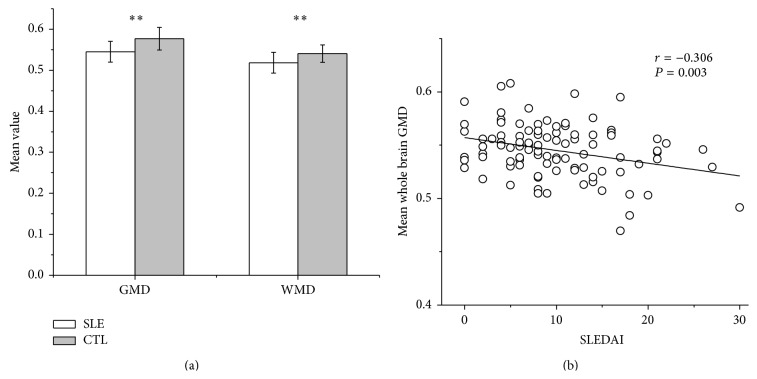
GMD/WMD reduction and the correlation between GMD with disease activity in non-NPSLE. (a) Significantly reduced GMD and WMD in SLE compared with CTLs; (b) negative correlation between GMD and the SLEDAI score of SLE. SLE: systemic lupus erythematosus; CTL: control; GMD: mean whole brain grey matter density; WMD: mean whole brain white matter density; SLEDAI: systemic lupus erythematosus disease activity index. ^*^ *P* < 0.05, ^**^ *P* < 0.01.

The GMD and WMD were compared between the SLE patients and CTLs. Both the GMD and WMD were significantly decreased in the SLE group compared with the CTL group (Table 1, Figure 1(a)).

### 3.4. Association between GMD/WMD and Symptomatic Severity

Comparison of the GMD/WMD between the active and inactive SLE patients showed a lower GMD in the active patients than the inactive patients. There was a negative correlation between the total SLEDAI score and GMD (*r* = −0.306, *P* = 0.003; Figure 1(b)). Considering the possible influence of age on grey matter, we carried out a partial correlation using age as the control variable to assess the correlation between the disease severity and GMD. The results demonstrated that the negative correlations between the total SLEDAI score and GMD persisted (*r* = −0.338, *P* = 0.001). No significant correlation was found between the SLEDAI score and WMD (*r* = −0.081, *P* = 0.437). There was also no significant correlation between the SLICC score and GMD (*r* = −0.153, *P* = 0.139) or WMD (*r* = −0.002, *P* = 0.987).

### 3.5. Association of GMD/WMD with Different Therapies

**Figure 2:**
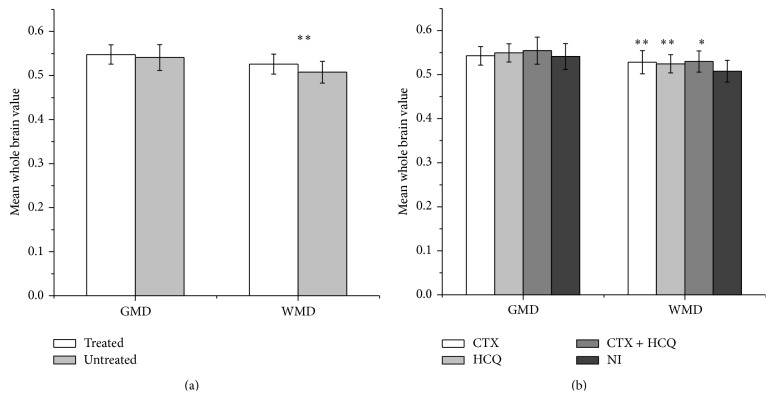
GMD/WMD and the relationships with different treatments in SLE patients. The patients receiving treatment with immunosuppressive agents had a greater WMD than the patients who were never treated with immunosuppressive agents. There was no significant difference in the GMD between the two groups (a). The CTX-, HCQ-, or CTX+HCQ- treated patients had significantly higher WMDs compared with the NI group. Pairwise-group comparisons showed that the NI patients had the lowest WMD (b). GMD: mean whole brain grey matter density; WMD: mean whole brain white matter density; CTX: cyclophosphamide; HCQ: hydroxychloroquine; NI: nonimmunosuppressive agents treated. ^*^ *P* < 0.05, ^**^ *P* < 0.01.

In this study, 47 patients were newly diagnosed with SLE. However, there was no significant difference in the GMD/WMD between the newly diagnosed and long-term patients. There was no association between the total corticosteroids and GMD or WMD (*r* = −0.032, *P* = 0.759 for GMD and *r* = −0.099, *P* = 0.338 for WMD). The possible effect of immunosuppressive agents on the brain structure was also considered. First, the 95 patients were divided into two groups. One group was treated with the immunosuppressive agents HCQ, CTX or both until the study day, including any current or previous treatment (treated group, *n* = 55). The other group was never treated with immunosuppressive agents (untreated group, *n* = 40). The treated group had a greater WMD (*t* = 3.793, *P* < 0.0001) than the untreated group (Table 2, Figure 2(a)). The GMD was not significantly difference between the two groups (*t* = 1.286, *P* = 0.202).

Of the 55 patients, 17 patients received CTX, 32 received HCQ, and 6 received CTX and HCQ. Thus, the 95 patients were then divided into four groups to further study the effect of the different therapies on the GMD and WMD. The four groups were the non-ISA treated (NI), CTX, HCQ, and CTX + HCQ groups. Considering the possible influence of age and severity of SLE on the GMD and WMD, we used age and the SLEDAI score as the control factors to perform covariance ANCOVA analysis. The results showed that there was a significant intergroup difference in the WMD (between-group *P* value = 0.003). The results of a pairwise-group comparison showed that the NI patients had the lowest WMD. The CTX-, HCQ-, and CTX+HCQ-treated patients had significantly higher WMD compared with the NI group (see Supplementary Table 1 in Supplementary Material available online at http://dx.doi.org/10.1155/2015/920718, Figure 2(b)). There were no significant differences in the GMD between four groups.

### 3.6. GMD/WMD and Autoantibodies

**Figure 3:**
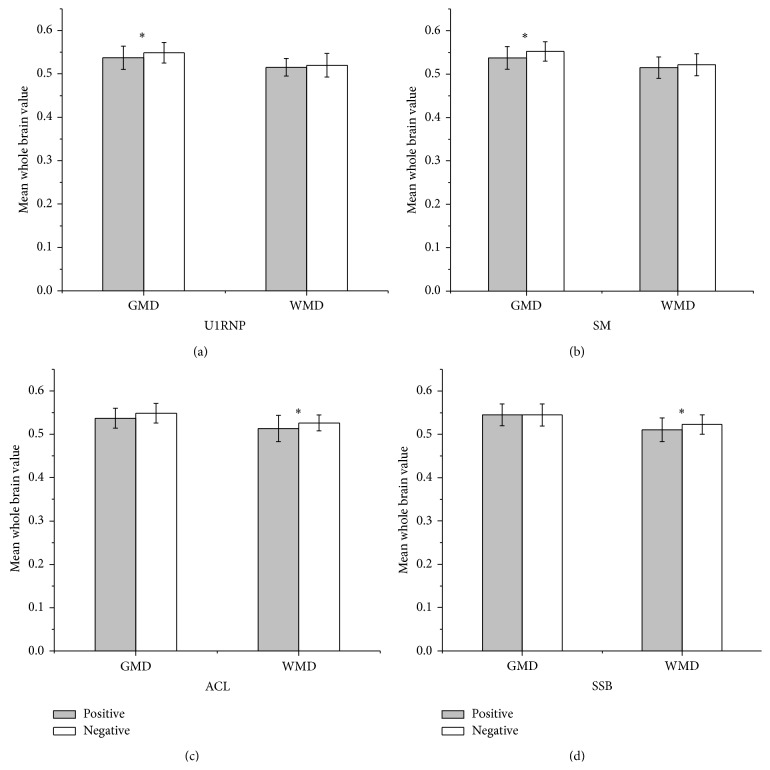
GMD/WMD difference and the relationships with different autoantibodies in SLE patients. The anti-U1RNP and anti-SM antibody-negative patients had higher GMDs than the antibody-positive patients (a, b). The ACL- and anti-SSB/La antibody-negative patients had higher WMDs than the antibody-positive patients (c, d). GMD: mean whole brain grey matter density; WMD: mean whole brain white matter density; ACL: anticardiolipin antibodies; SSB: anti-SSB/La antibodies; U1RNP: anti-U1 RNP antibodies; SM: anti-Sm antibodies. ^*^ *P* < 0.05.

Ten autoantibodies were detected in all 95 patients. Among all of the patients, 33 patients were ACL-positive (34.74%). The GMD of the anti-U1RNP antibody-positive patients was significantly reduced compared with the antibody-negative patients (*t* = −2.095, *P* = 0.039). A similar result was found for the anti-SM positive and negative patients, with the anti-SM positive patients showing a significantly reduced GMD compared to the antibody-negative patients (*t* = −2.938, *P* = 0.004) (Table 3, Figure 3). There was a possible trend toward a lower GMD in the antihistone antibody-positive patients (*t* = −1.934, *P* = 0.056).

We found a significant difference in the WMD between the ACL positive and negative patients groups (*t* = −2.186, *P* = 0.032). The anti-SSB/La-positive patients also had a greater reduction in the WMD than the anti-SSB/La-negative patients (*t* = −2.313, *P* = 0.023). There was a possible trend of a lower WMD in the anti-SSA/Ro52 kD antibody-positive patients, but this was not significant (*t* = −1.936, *P* = 0.056). For the other three antibodies, anti-P0, anti-dsDNA, and anti-SSA/Ro60 kD, there were no significant differences in the GMD and WMD between the antibody-positive and -negative patients.

Negative correlations between the ANA and GMD and WMD were found (*r* = −0.241, *P* = 0.019 for both GMD and WMD). However, when we controlled for age, these trends disappeared (*r* = −0.125, *P* = 0.302 for GMD and *r* = −0.205, *P* = 0.089 for WMD).

## 4. Discussion

In this study, we found a clear reduction in the GMD and WMD in the NPSLE patients. These patients were previously identified as normal by conventional MRI. We also identified relationships between several autoantibodies and GM and WM reduction in the NPSLE patients. The presence of these specific autoantibodies might help identify early brain damage in NPSLE patients.

Although MRI is considered to be a good method to evaluate CNS manifestations in SLE, conventional or anatomical MRI findings are often nonspecific or negative in patients with and without NPSLE. The patients in this study did not have major CNS manifestations or disease; therefore, the GM and WM loss implied that brain damage occurs before clear clinical neurological symptoms are present. Consistent with a previous magnetic resonance spectroscopy (MRS) study, our results supported the notion that abnormal microstructural changes may occur before the appearance of any clear CNS symptoms or conventional imaging signatures [8]. Here, we applied a more advanced method that can calculate the precise quantitative whole-brain GMD and WMD. Thus, the present findings also highlight the value of quantitative volumetric MRI techniques in detecting minor GM and WM reductions in NPSLE. This may aid in predicting NPSLE in patients and better reporting the cumulative injury inflicted by SLE.

Until now, the role of antibodies in the pathophysiology of brain damage in SLE was unclear. Our results are the first to identify the relationship between GMD/WMD and autoantibodies. Anti-U1RNP and anti-SM antibodies demonstrated greater effects on the GMD, while ACL and SSB showed greater effects on the WMD. Anti-U1RNP antibodies may be linked to central neuropsychiatric manifestations [18] and act as an inducer of proinflammatory cytokines. In a previous study, a correlation between the presence of anti-Sm antibodies in the serum and central NPSLE was observed [19]. The anti-Sm autoimmune response is a polyclonal humoral immune response against protein components of small nuclear ribonucleoprotein (snRNP) particles and is found in greater than 30% of the patients with SLE. This response is specific to SLE [20]. The association between anti-U1RNP and anti-SM antibodies with the GMD reduction suggests a possible diagnostic and prognostic value of these antibodies in determining CNS involvement in SLE. Various autoantibodies, including ACL, have been implicated in the pathogenesis of NPSLE [21]. ACL has been a focus in SLE research [22] and was reported to be associated with neuropsychiatric manifestations [10] and brain abnormalities [23]. As phospholipids are the main constituent of WM, we evaluated ACL and found a reduction of the WMD in ACL-positive patients. Another study using magnetization transfer imaging also found an association between the presence of ACL and cerebral damage in grey and white matter in NPSLE [24]. In combination with these studies, our results provide evidence that ACL might damage WM even before patients show obvious neuropsychiatric symptoms. Because of their prothrombotic tendency [25], ACLs may cause cerebral ischemia and result in white matter atrophy. The anti-SSB/La antibodies were also related to the observed WMD reduction. The exact role of anti-SSB/La pathology in WM damage is unclear. It has been reported that anti-SSB/La antibodies can cause increased neutrophil apoptosis and decreased phagocytosis and affect the inflammation process [26]. Thus, it is possible that the abnormal inflammatory reactions [27] or the secondary reactions in SLE can induce WM atrophy.

Our results implicate other antibodies, specifically the anti-P0, anti-SSA/Ro60 kD, antinucleosome or anti-dsDNA antibodies, in CNS. The remaining tested antibodies showed only possible associations with the GMD and WMD reduction, such as antihistone antibodies with GMD and anti-SSA/Ro52 kD antibodies with WMD. The role of antibodies in the pathology of SLE may be complex and the results sometimes contradictory. For example, anti-P antibodies recognize specific proteins on ribosomes, and anti-P antibodies detected in blood have been associated with psychosis in some studies [28]; however, these possible associations have not been confirmed in other studies [29]. Precise and prospective cohort studies that discuss the association between these antibodies and brain damage, including both grey matter and white matter, are needed.

This study also found that the patients treated with immunosuppressive agents (CTX, HCQ, or both) had increased WMD compared with the patients who were never treated with immunosuppressive agents. This finding suggests a potential protective role of immunosuppressive agents in preventing WM atrophy. Several studies support using CTX in the treatment of NPSLE [30]. The potential neuroprotective effect of CTX has been identified in SLE [31] and other white matter demyelinating diseases, such as antiphospholipid syndrome [32] and experimental autoimmune gray matter disease [33]. A possible mechanism for the neuroprotective effect of immunosuppressive agents may be their ability to reduce demyelination due to vasculitis. However, further studies are needed to determine the advantages and disadvantages of long-term immunosuppressive therapy. On the other hand, the protective effect of immunosuppressive therapy was more obvious in the WMD. This result might imply that WM is more sensitive than GM to immunosuppressive agents. Early use of immunosuppressive agents may prevent the aggressive reduction of brain density.

The loss of GM and WM may originate from the brain atrophy previously described in SLE [5, 34, 35]. However, the exact mechanism of brain atrophy in SLE remains unclear. The WM hyperintensity in SLE that has been demonstrated in longitudinal research may become progressive over time in patients with severe SLE [36] and may be caused by the neurotoxic effect of the chronic disease. There are several possible explanations for the atrophy: (1) neurodegenerative changes due to axonal damage that is primary or secondary to the vasculopathy in SLE; (2) some antibodies, such as APL, affecting both the small vascular and brain cellular elements that lead to cerebral dysfunction [36] are reportedly related to nervous system damage similar to that seen in NPSLE [24]; (3) the activation of a cytokine network independent of the pathological process has been observed in SLE patients with CNS complications, which suggests a neurotoxic effect of cytokines in SLE [37]; (4) damage of the brain endothelium causes damage to the blood-brain barrier, which normally restricts the entry of plasma constituents, including proteins [38]; (5) demyelination originates from decreased serum brain-derived neurotrophic factor (BDNF) levels in patients [39].

As an invasive technique, MRI is considered to be a useful tool in evaluating the involvement of the CNS in SLE [4, 40]. This study has provided evidence for microstructural brain atrophy preceding the emergence of a clear neurological manifestation in NPSLE. Because the patients in our study were all without obvious neuropsychiatric symptoms or obvious structural abnormalities detected by conventional MRI, the results presented here support brain involvement as a primary deficit in SLE and suggest the neuroprotective effect of immunosuppressive therapy in managing white matter atrophy. We believe that these results might have significant value in the early diagnosis of CNS involvement in SLE. On the other hand, the tight relationship between autoantibodies and microstructural brain damage reveals the value of brain microstructural damage as one of the sensitive indicators for the CNS involvement in SLE. There are several limitations of this study. Although we used a quantitative method to calculate the GMD/WMD, the segmentation of grey matter and white matter based on the MRI signal does not reflect the pathology of the delineation of grey matter and white matter or the exact pathological mechanisms of the GMD/WMD reduction, such as atrophy, apoptosis, or demyelination. Future studies are needed to help us understand the underlying complex mechanisms of CNS deficit in SLE.

## Supplementary Material

Pairwise-group comparisons showed that the NI patients had the lowest WMD in four groups. The CTX-, HCQ- and CTX+HCQ- treated patients had higher WMD compared with the NI group. There were no significant differences in the GMD between four groups.

## Figures and Tables

**Table 1:** Demographic and clinical characteristics of SLE patients and healthy controls.

	Group		*t*	*P*
	SLE (*n* = 95)	CTL (*n* = 89)		
Age (year, mean ± SD)	28.65 ± 7.51	30.70 ± 7.93	−1.795	0.074
Female/male	79/16	64/25	3.357 (*x* ^2^)	0.067
Duration (month, mean ± SD)	18.99 ± 27.55	NA		
SLEDAI (mean ± SD)	10.01 ± 6.45	NA		
Total steroid (g, mean ± SD)	6.86 ± 12.05	NA		
Total CTX (g, mean ± SD)	0.96 ± 0.24	NA		
Total HCQ (g, mean ± SD)	23.19 ± 66.22	NA		
GMD	0.5450 ± 0.0253	0.5767 ± 0.0276	−8.132	0.000
WMD	0.5183 ± 0.0252	0.5405 ± 0.0213	−6.455	0.000
Manifestation (*n* (%))				
Seizure	0 (0)			
Psychosis	0 (0)			
Organic brain syndrome	0 (0)			
Visual disturbance	0 (0)			
Cranial nerve disorder	0 (0)			
Lupus headache	0 (0)			
Cerebrovascular accident (CVA)	0 (0)			
Neurological sign	0 (0)			
Vasculitis	5 (5.26)			
Arthritis	25 (26.32)			
Myositis	5 (5.26)			
Urinary casts	2 (2.11)			
Hematuria	30 (31.58)			
Proteinuria	24 (25.26)			
Pyuria	32 (33.68)			
Malar rash	25 (26.32)			
Discoid rash	8 (8.42)			
Photosensitivity	18 (18.95)			
Alopecia	18 (18.95)			
Mucosal ulcers	6 (6.32)			
Pleurisy	10 (10.53)			
Pericarditis	5 (5.26)			
Fever	4 (4.21)			
Low complement	79 (83.16)			
Thrombocytopenia	9 (9.47)			
Leukopenia	30 (31.58)			
Autoantibody positive (*n* (%))				
Antinuclear	95 (100)			
ACL	33 (34.74)			
Histone	51 (53.68)			
P0	46 (48.42)			
SM	45 (47.37)			
dsDNA	57 (60.00)			
SSA52	51 (53.68)			
SSA60	61 (64.21)			
SSB	34 (35.79)			
U1RNP	30 (31.58)			
Nucleosome	38 (40.00)			

SLEDAI: SLE disease activity index; GMD: mean whole brain grey matter density; WMD: mean whole brain white matter density; CTX: cyclophosphamide; HCQ: hydroxychloroquine; CTL: healthy control; ACL: anticardiolipin antibodies; histone: antihistone antibodies; P0: antiribosomal P antibodies; SM: Anti-Sm antibodies; ds-DNA: anti-dsDNA antibodies; SSA52: anti-Ro/SSA 52-KD antibodies; SSA60: anti-Ro/SSA 60-KD antibodies; SSB: anti-La/SSB antibodies; U1RNP: anti-U1 RNP antibodies; nucleosome: antinucleosome antibodies; NA, not applicable.

**Table 2:** Comparison of GMD/WMD.

	GMD		*t*	*P*	WMD		*t*	*P*
	Mean	SD			Mean	SD		
SLE (*N* = 95)	0.5450	0.0253	−8.132	0.000^**^	0.5183	0.0252	−6.455	0.000^**^
CTL (*N* = 89)	0.5767	0.0276			0.5405	0.0213		
Treated (*N* = 55)	0.5496	0.0221	1.286	0.202	0.5262	0.0224	3.868	0.000^**^
Untreated (*N* = 40)	0.5388	0.0282			0.5074	0.0248		
Active ( *N* = 49)	0.5399	0.0266	−2.069	0.041^*^	0.5169	0.0280	−0.555	0.580
Inactive (*N* = 46)	0.5505	0.0328			0.5198	0.0220		
First diagnosis (*N* = 47)	0.5413	0.0267	−1.425	0.158	0.5154	0.0274	−0.233	0.273
Long duration (*N* = 48)	0.5487	0.0236			0.5211	0.0226		

GMD: mean whole brain grey matter density; WMD: mean whole brain white matter density; ^*^ *P* < 0.05, ^**^ *P* < 0.01.

**Table 3:** GMD/WMD comparison between autoantibody-positive and -negative patients.

	AB-positive patient		AB-negative patient		*t*	*P*
	Mean	SD	Mean	SD		
U1RNP	*N* = 30		*N* = 65			
GMD	0.5372	0.0272	0.5487	0.0237	−2.095	0.039^*^
WMD	0.5152	0.0202	0.5197	0.0271	−0.805	0.423
Sm	*N* = 45		*N* = 50			
GMD	0.5373	0.0263	0.5520	0.0223	−2.938	0.004^**^
WMD	0.5147	0.0247	0.5215	0.0253	−1.321	0.190
ACL	*N* = 33		*N* = 39			
GMD	0.5372	0.0227	0.5483	0.0226	−1.894	0.062
WMD	0.5135	0.0301	0.5261	0.0184	−2.186	0.032^*^
SSB	*N* = 34		*N* = 61			
GMD	0.5452	0.0252	0.5449	0.0255	0.049	0.961
WMD	0.5105	0.0277	0.5226	0.0227	−2.313	0.023^*^
SSA52	*N* = 51		*N* = 44			
GMD	0.5464	0.0243	0.5434	0.0266	0.564	0.574
WMD	0.5137	0.0257	0.5236	0.0238	−1.936	0.056
SSA60	*N* = 61		*N* = 34			
GMD	0.5471	0.0235	0.5413	0.0281	1.070	0.288
WMD	0.5162	0.0246	0.5221	0.0261	−1.110	0.270
Histone	*N* = 51		*N* = 44			
GMD	0.5404	0.0213	0.5504	0.0228	−1.934	0.056
WMD	0.5134	0.0254	0.5225	0.0244	1.781	0.078
P0	*N* = 46		*N* = 49			
GMD	0.5456	0.0271	0.5445	0.0237	0.218	0.828
WMD	0.5169	0.0276	0.5196	0.0228	−0.510	0.611
Nucleosome	*N* = 38		*N* = 57			
GMD	0.5418	0.0279	0.5472	0.0234	−1.019	0.311
WMD	0.5200	0.0251	0.5171	0.0253	0.549	0.584
DsDNA	*N* = 63		*N* = 32			
GMD	0.5434	0.0274	0.5483	0.0206	0.899	0.371
WMD	0.5153	0.0233	0.5241	0.0279	1.613	0.110

AB: antibodies; ACL: anticardiolipin antibodies; histone: antihistone antibodies; P0: antiribosomal P antibodies; SM: Anti-Sm antibodies; ds-DNA: anti-dsDNA antibodies; SSA52: anti-Ro/SSA 52-KD antibodies; SSA60: anti-Ro/SSA 60-KD antibodies; SSB: anti-La/SSB antibodies; U1RNP: anti-U1 RNP antibodies; nucleosome: antinucleosome antibodies; ^*^ *P* < 0.05, ^**^ *P* < 0.01.
