# Supplementary material for: Autoantibodies Affect Brain Density Reduction in Nonneuropsychiatric Systemic Lupus Erythematosus Patients
Source: J Immunol Res. 2015 May 18;2015:920718. doi: 10.1155/2015/920718 (PMC4451776; doi:10.1155/2015/920718)
Supplement: Supplementary file 1 — Pairwise-group comparisons showed that the NI patients had the lowest WMD in four groups. The CTX-, HCQ- and CTX+HCQ- treated patients had higher WMD compared with the NI group. There were no significant differences in the GMD between four groups. [file 920718.f1.pdf]

Supplementary Table 1 GMD/ WMD of SLE patients with different therapies.

	group								Comparison		
	CTX N=17		HCQ N=32		CTX+HCQ N=6		NI N=40		CTX: NI	HCQ: NI	CTX+HCQ: NI
	Mean	SD	Mean	SD	Mean	SD	Mean	SD	P		
GMD	0.5427	0.0210	0.5494	0.0207	0.5545	0.0308	0.5411	0.0292	0.790	0.730	0.133
WMD	0.5281	0.0266	0.5243	0.0206	0.5299	0.0243	0.5076	0.0247	0.004**	0.004**	0.029*

GMD: mean whole brain grey matter density; WMD: mean whole brain white matter density; CTX: cyclophosphamide; HCQ: hydroxychloroquine; NI: non-immunosuppressive treatment; *p<0.05， ** p<0.01
